# The health system and access to treatment in patients with cervical cancer in Mexico

**DOI:** 10.3389/fonc.2022.1028291

**Published:** 2022-11-30

**Authors:** Eder A. Arango-Bravo, Lucely del Carmen Cetina-Pérez, Tatiana Galicia-Carmona, Denisse Castro-Eguiluz, Dolores Gallardo-Rincón, Ivette Cruz-Bautista, Alfonso Duenas-Gonzalez

**Affiliations:** ^1^ Department of Clinical Research, Instituto Nacional de Cancerología, Mexico, Mexico; ^2^ Integral program for the care of locally advanced and metastatic cervical cancer (MICAELA), Instituto Nacional de Cancerología, Mexico, Mexico; ^3^ Department of Medical Oncology, Instituto Nacional de Cancerología, Mexico, Mexico; ^4^ Department of Oncology, Hospital Médica Sur, Mexico, Mexico; ^5^ Investigador por México CONACYT- Department of Clinical Research, Instituto Nacional de Cancerología, Mexico, Mexico; ^6^ Department of Metabolic Diseases Research, INCMNSZ, Mexico, Mexico; ^7^ Instituto de Investigaciones Biomedicas, Universidad Nacional Autonoma de Mexico, Mexico, Mexico; ^8^ Division of Basic Research, Instituto Nacional de Cancerologia, Mexico, Mexico

**Keywords:** cervical cancer, Mexican healthcare system, HPV vaccination, pap smear, oncological resources

## Abstract

Cervical cancer (CC) is tightly related to a low Human Development Index. Mexico is an upper-middle-income country with 126 million inhabitants, and its public health system aims to provide universal health coverage. Currently, employment-based social insurance covers approximately 60% of the population, and the scope of the remaining 40% is on course *via* the “IMSS-Bienestar” Institute. However, the annual government spending on health remains at 3% of the Gross Domestic Product, which is well below the 6% recommended by the Organization for Economic Cooperation and Development. CC is the second in incidence and mortality among women. Regarding primary prevention with the Human Papilloma Virus-vaccine, the current coverage for girls aged 9 to 14 years is only around 7%. Among secondary prevention with screening, the program is yet to cover the total number of women at risk; nevertheless, the age-standardized CC mortality rate has decreased from 12 per 100,000 women in 1979 to 5.7 per 100,000 women in 2020 due in part to increased screening coverage. Still, around two-thirds of patients present with locally advanced disease at diagnosis. Data from our country demonstrate that even socially disadvantaged CC patients achieve “standard” survival outcomes if treatment is granted. Nevertheless, there is a shortage in almost every aspect regarding CC treatment, including oncologists, chemotherapy units, medical physicists, radiation technicians, and both teletherapy and brachytherapy facilities. In conclusion, advances in the public health system in Mexico are urgently required to achieve CC control and reduce the mortality from this neoplasia that mainly targets socially disadvantaged women.

## Global burden of cervical cancer

Cervical cancer (CC) is the fourth most incident cancer and the fourth cause of death by cancer in women, with approximately 604,000 new cases and 342,000 deaths worldwide by 2020. In addition, it is the most diagnosed malignant disease in 23 countries and the leading cause of death in 36 countries. Most of these are in sub-Saharan Africa, Melanesia, South America, and South-Eastern Asia (1).

The Human Development Index (HDI) is strongly negatively associated with CC incidence and mortality. The rates in developed and developing countries vary from 18.8 vs. 11.3 per 100,000 and 12.4 vs. 5.2 per 100,000 for incidence and mortality, respectively. The difference occurs even within high-income countries such as the United States of America (USA), where the death rate from CC is twice as high among women living in high poverty than those in low-poverty areas (1, 2).

## Cervical cancer in Mexico

Mexico is an upper middle-income country (UMIC) in the current World Bank classification of countries by income. Mexico had a population of 126,014,024 inhabitants in 2020. The adjusted incidence of CC in 2020 was 12.6 per 100,000 women. Data from the National System of Statistical and Geographical Information (INEGI), which registers mortality, indicates that CC mortality rates have decreased from 12 to 5.7 per 100,000 from 1979 to 2020. Still, this neoplasia represents the second cause of cancer in Mexican women, with 9,439 new cases per year, and the second cause of death, with 4,335 cases. Among women with invasive CC, around 70% are diagnosed with locally advanced disease. These figures speak on deficiencies in coverage and timely diagnosis and treatment of detected preinvasive and invasive lesions (2–4).

Information regarding the number of CC patients attended at major public institutions is scarce. A retrospective study that included 346 women diagnosed with CC from an Oncology Center showed that 65.32% of patients were stage II and III according to the International Federation of Gynecology and Obstetrics (FIGO, 2009) (5). Likewise, a third-level hospital of another Oncology Center reported that of 111 patients, 76.4% were in stages II and III (6). In 2020, a cohort of 2,982 women diagnosed with CC treated at the National Cancer Institute of Mexico (Incan) from 2005 to 2015 was reported. The study showed that most patients were diagnosed with locally advanced disease (1B2 –IVA, FIGO), 73.10% in women younger than 40 and 78.1% in women older than 40. Early disease (IA1 –IB1, FIGO) represented 15% of women younger than 40 years compared to 19.3% in those older than 40 years, and advanced disease (IVB, FIGO) corresponded to 7.58% in young women compared to 6.93% in older women (7). Recently, in 2022, a retrospective analysis was published that included more than 20,000 patients diagnosed with CC, whose treatment was financed by the Popular Insurance Catastrophic Expenditure Protection Fund (FPCGC). The prevailing clinical stage at the time of diagnosis was locally advanced disease (FIGO) in 14,782 women (68.5%), followed by early disease in 5,286 patients (24.5%) and advanced disease in 1488 women, corresponding to 6.9% (8). Among 346 CC patients treated at an oncology hospital, more than half of the women did not have a formal job (57%), two-thirds of the women had social security through a family member or their retirement, and 32% had social security coverage through their employment. Nine percent of these women were illiterate, and most did not complete middle school (77%) (5).

## Public health system of Mexico

In Mexico, article 4 of the Mexican Political Constitution, amended in May 2020, establishes: “*…every person has the right to health protection.”* Accordingly, the health system in Mexico is public, intended to provide medical care to all, and it is currently transitioning to accomplish what is written by Law. Up to now, the Mexican public health system has two main components operating in parallel:

1) Employment-based social insurance schemes. These include 1.1. The Mexican Institute of Social Security (IMSS), 1.2. The Institute of Social Security and Services for State Workers (ISSSTE), 1.3. The Social Security Institution of Federal Entities (ISSES), 1.4. The employee of the Mexican Petroleum Public company (PEMEX), and 5.1. The Social Security Institute of the Armed Forces (ISSFAM).

2) The Population with no Social Security Services, which several public funds serve. 2.1. The Federal Entities Spending on Health (Field 12 from Health Secretary), 2.2. The Fund for Health Services (Field 33-FASSA), 2.3. The IMSS-Bienestar, 2.4. The Armed Forces Secretary (SEDENA), and 2.5. The Marine Secretary (SEMAR). Accordingly, the public spending on health by the Mexican government is channeled to 5 institutions of the Employment-based subsystem and 5 Institutions of the Population with no social security services. According to the 2020 data from the INEGI, Mexico has a total population of 126,014,024 million, and the percentages of public insurance are as follows:

1. Employment-based social insurance schemes: IMSS 51%, ISSSTE, and ISSES 8.8%, PEMEX and ISSFAM 1.3%. This subsystem covers 61.1% of the population.
2. Population with no Social Security Services served by several public funds: INSABI 35.5%, IMSS-Bienestar 1%, others 1.2%, covering 37.7% of the population.

Thus, in theory, the total population covered by public health services in Mexico is almost 100%. However, the actual coverage for people with no Employment-based Social Security Services (37.7%) is yet to occur. Currently, the government is reorganizing the IMSS-Bienestar Institute to make this subsystem the primary public health Institution to cover every individual lacking Employment-based social security (9, 10).

As in many countries, the private health sector is operating as well. The INEGI 2020 data discloses that 2.8% of the population has private insurance, mostly individually contracted and also granted by some private companies to their employees (11). It also must be noticed that many pharmacy chains throughout the territory sell medicines and have a general practitioner physician consultation service for free or a small fee. This system of pharmacies with their primary care physicians represents an affordable option for a population segment (with or without access to public health services). Some people with access to public health services would prefer to pay a relatively small fee than wait in long lines to access their public health clinic that does not always have medicines in stock. Of course, this system works only for relatively simple health issues that do not require hospitalization or a specialized level of care. The overall impact of this private subsystem on the government’s public health service remains to be determined (12).

This work does not intend to analyze the Mexican Public Health system deeply. Still, for any informed citizen, the public health system in Mexico has two fundamental flaws that, when combined, explain why it is deficient. The insufficient public resources allocated and its fragmentation into several subsystems. From a comparative perspective, resources allocated to public health by the Mexican government fall well below the spending average of the countries of the Organization for Economic Cooperation and Development (OECD) and of international recommendations, which, according to the World Health Organization (WHO), it should be 6% of the Gross Domestic Product (GDP). In 2019, the OECD countries spent, on average, 6.6%, while public spending on health by the Mexican State represented only 2.7% of GDP. This data implies a per capita expenditure of 555 USD, which places the country well below the OECD average (3,040.55 USD).

Regarding private expenses as a percentage of the GDP, the average for countries of OECD was 2.2% (6.6% public, 2.2% private, a total of 8.8%). In Mexico, the average personal expense was 2.8%, similar to the public expense of 2.8% for a total of 5.6%. The current perspectives on the public expense on health are not very encouraging. Between the years 2004-2021, the average was 2.86%. The lowest was 2.5% in 2005 and 2006, and the highest was 3.1% in 2009, 2010, 2011, 2012, and 2013. The estimates for 2020 and 2021 were 2.9%. The expending for the Employment-based systems and those services for the population with no Social Security Services remained the same at 1.7% and 1.2%. Thus, despite the Law that states that all individuals must have medical service coverage, many do not have it, or if they do, it is suboptimal. These figures on federal spending on health have dramatic consequences. The availability of health resources such as beds, medicines, medical supplies, and health professionals (medical and nurses) is poor—for example, the ratio of beds and staff doctors for every thousand inhabitants. In Mexico, we had only 1.8 beds per thousand inhabitants in 2000, a figure well below the 4.5 beds per every thousand in OECD countries. Regarding medical personnel in Mexico, there are only 2.4 doctors for every thousand inhabitants, compared to the OECD countries with 3.6. With this scenario on public health, it is not surprising that the oncology infrastructure is also deficient (10).

A recent report in Mexico establishes that most cancer services (81%) were delivered by the public while 19% by the private sector (13). The numbers of specialized cancer units are 118 establishments in total. Of these, 65 are public, 48 are private, and 5 are mixed. Regarding equipment for diagnosis, 31 positron emission tomography-computed tomography (PET-CT) equipments, 793 Computed Tomography (CT) scanners, and 316 magnetic resonance imaging (MRI) equipments are available (14). The IMSS alone which is the main employment-based social insurance has medical units for the first, second, and third level of care. The first level units are located throughout the national territory, and most preventive actions are related to the timely detection of breast cancer and CC. From the third level units, it has only 20 specialized centers providing oncological care (15).

According to the Mexico Radiation Oncology Certification Board, a national census revealed Mexico’s infrastructure and radiotherapy (RT) units. One hundred and three RT centers were documented. These centers contain a total of 162 RT machines, 141 linear accelerators, and 21 radionuclide therapy units—19 are teletherapy cobalt-60 (90.5%) and 2 radionuclide stereotactic units (9.5%), both GammaKnife. This data represents a median of 3 machines by federal entity (except in Tlaxcala, which has no radiotherapy, and 46 are located in Mexico City). Eighteen federal entities have less than 3 machines (56.25%). The total density of RT machines per million inhabitants is 1.32, ranging from 0 in Tlaxcala to 5.16 in Mexico City. Of the 103 RT centers, 59 (57.3%) have brachytherapy units (median of 1 center with brachytherapy units by state). Five states have no brachytherapy units (15,6%), 11 states have 1 unit (34,4%), 8 states have 2 units (25%), 5 states have 3 units (15,6%), and 1 state has up to 15 units (3,1%). The global rate of brachytherapy units per million inhabitants is 0.55. Thirty-seven brachytherapy units (56.1%) use automated high-rate dose, and 29 units (43.9%) use low-rate dose (16). Mexico stands last with only 1.3 RT machines per million inhabitants, while there are 18.7 for Switzerland and 11.3 for the USA.

Regarding cancer specialists, there were 945 surgical oncologists for adults and 24 surgical oncologists for children, 473 medical oncologists, 174 gynecological oncologists, and 264 pediatric oncologists. In a list of selected countries, the USA heads with 161 oncologists per million inhabitants, followed by the United Kingdom and Italy with 131 and 122, while Mexico has only 16 per million (17). The report from the Mexico Radiation Oncology Certification Board stated that since 1988, 368 radiation oncologists had been certified. Of these, 346 remain active in oncologic institutions. This fact translates into 1 radiation oncologist per 345,000 inhabitants (16). Altogether, these data indicate that Mexico’s public health system cannot provide coverage to the whole population promptly and efficiently. Much work needs to be done to increase government spending on health and, at the same time, to be organized in a centralized manner to optimize the scarce existing resources.

## Primary prevention in Mexico (HPV vaccination)

In Mexico, vaccination against HPV was first introduced in 2008 with low coverage to girls aged 12-16 years using a 0 to 6-month schedule. One year later, an extended dosing schedule was introduced to target girls aged 9-12 for the first 2 doses, applied 6 months apart, followed by a third dose 60 months later. The vaccine was included in the national vaccine program until 2012. The coverage has increased over time; according to the last reported data in 2018, about 1 million doses were applied in Mexico (18). However, this number is still meager (around 7%) considering the population of 126 million, from which 5.7% are females between 9 and 14 years old (19). In this regard, Mexico faces, like many other countries with limited resources for public health, many obstacles to implementing vaccination schedules. Those barriers are multifactorial and include limitations in costs, infrastructure, and even social stigma (20). Due to these difficulties, the prevalence of HPV infections remains high in Mexico. Mexico is a region with a high rate of HPV infection (21, 22). Moreover, a high prevalence of HPV in women younger than 25 that attend college is likely related to risky sexual behavior, lack of knowledge of HPV infection, and other cultural factors (23). Because of that, even in the best scenario, the prospects for reducing CC mortality *via* primary prevention are discouraging.

## Secondary prevention in Mexico (screening)

In Mexico, despite historical efforts, CC continues to represent a high burden of cancer. The first actions for the timely detection of CC were implemented at the General Hospital of Mexico in 1974. In 1994, the official Mexican standard OM-O 14-SSA2-1994 for Prevention, Treatment, and Control of CC and Breast Cancer was established. According to the Norm, the Papanicolaou would have to be performed annually, and women whose cytology diagnosis was compatible with HPV infection would be referred to a colposcopy service. By then, it was not known that there was no treatment for HPV infection in the absence of lesions. Also, referring a woman with a morphological image suggestive of HPV infection to a colposcopy clinic unnecessarily increases costs and carries other issues like overdiagnosis and negative psychological consequences in women. In 1998 it was decided that the frequency of cervical cytology would be every three years in women with two consecutive annual negative results for HPV infection, dysplasia, or cancer; while women positive for HPV infection or dysplasia would be followed up in the clinic. After being discharged, they would start the annual periodicity again. On the other hand, women with positive results for nonspecific inflammatory processes should continue with annual exams until they have two consecutive negative results. In 2007, the Modification to the Mexican Official Standard (NOM-O 14-SSA2-1994) for the Prevention, Detection, Diagnosis, Treatment, Control, and Epidemiological Surveillance of CC privileged CC detection in women residents of rural and indigenous areas and marginalized urban areas (24–26).

Based on these experiences, in 2009, the norm incorporated vaginal self-sampling for high-risk HPV DNA testing. The main difficulty with this method lies in achieving, once the self-collection is done, that the sample arrives at a trained laboratory, that the sample is analyzed and that the results return promptly to the place of origin, where trained personnel must come to provide treatment and follow up on each case. Again, the main obstacle lies in the scarce availability of resources in marginalized areas. It was assumed that the incorporation of the high-risk HPV DNA test as a diagnostic complement to Pap smear could help reduce inequity in the quality of detection, modernize prevention and control strategies, increase coverage –without losing certainty in detection—and expand the detection coverage in areas with difficult access to health services. However, this can only be achieved if the institutional responsibilities in each case are precisely defined and fully adopted by the health services (27). They must ensure that the samples reach the laboratories, that they will be processed and sent promptly to those responsible for the treatment and follow-up of the patients, and that women can be treated appropriately. Though existing resources have recently increased, including regional molecular laboratories in several Mexican states, a pending issue is the lack of an integrated information system for accurate data on CC.

Currently, the fragmented information causes inaccurate epidemiological information. It makes it challenging to cross-reference information to understand better the problem, including the lack of data incorporation from private medical units. Consequently, there is only a partial diagnosis of the problem, which affects the design of programs and the allocation of resources to address them. Despite all these caveats, some progress has been made. The age-standardized CC mortality rate in Mexico in 1979 was 12 per 100,000 women, and the estimates for 2020 were 5.7 per 100,000. According to the current program (28), further progress can be expected if the program is better organized and adequately funded to increase coverage while reducing the high proportion of women lost to follow-up who do not receive treatment for their cervical lesion.

## Tertiary prevention in Mexico (treatment)

CC treatment is determined by clinical staging. Early-stage CC (IA1 to IB1) is primarily surgically treated. Locally advanced disease (IB2 to IVA) is treated with cisplatin-based concurrent chemoradiation followed by brachytherapy (either low-rate or high-rate dose). Advanced disease (IVB) is usually treated with carboplatin-paclitaxel doublet chemotherapy. Bevacizumab is only employed in selected patients (29). The lack of a cancer registry at the National level in Mexico is a severe drawback to having reliable epidemiological data on percentages of invasive CC patients regarding the FIGO clinical stage at presentation. Likewise, no information exists at the national level on the percentage of patients that receive optimal care. Available data are summarized in **Table 1**. Recent data from the ISSSTE informs that only 1.8% of the patients underwent surgery as a single modality; 6.7% underwent surgery plus adjuvant cisplatin-based concurrent chemoradiation; and 51.8% received definitive treatment with cisplatin-based concurrent chemoradiation, of which 77.6% completed treatment with brachytherapy. The most common treatment modality was radiotherapy alone in 28%; surgery followed by radiotherapy in 10%; 6% in the advanced disease subgroup received bevacizumab in combination with chemotherapy. The following drugs are available in this institution for managing locally advanced, recurrent, or metastatic disease: carboplatin, cisplatin, capecitabine, docetaxel, 5-fluorouracil, gemcitabine, and bevacizumab (6).

**Table 1:** Summary of resources for diagnosis and treatment of Cervical Cancer in Mexico.

Diagnosis			Treatment				
Imaging equipment (number of units in Mexico)(15)	Clinical Stage	Patients diagnosed at each clinical stage (8)	Modality (31)	Oncologists (number) (17,18)	Infrastructure (17)	Pharmacological treatment (6)	Five year-Overall survival (8)
Computed tomography scanners (793) Positron emission tomography-Computed Tomography (31) Magnetic resonance imaging (316)	Early Diasease	24.5%	Surgery Surgery and adjuvant radiation or adjuvant chemoradiation	Surgical oncologists (945); Gynecological oncologists (174)		Cisplatin	88%
	Locally advanced Disease	68.5%	Concurrent chemoradiation and brachytherapy	Medical oncologists (473); Radiation oncologist (346)	Teletherapy equipments (162); brachytherapy units (59)	Cisplatin Gemcitabine Carboplatin	63.9%
	Advanced Disease	6.9%	Doublet chemotherapy with/without bevacizumab	Medical oncologist (473)		Cisplatin Carboplatin Paclitaxel Bevacizumab 5-Fluorouracil Docetaxel Capecitabine	43.6%

It can be inferred from the shortage in oncological infrastructure that not all CC patients are treated effectively and on time. Despite those caveats, it can be suggested that the outcomes of patients in Mexico treated for invasive CC are within the expected, according to a recent study (8). To place this study in perspective, currently, the population with no Employment-based Social Security Services in Mexico is 37.7%, and the goals of the current Federal Administration are to cover this population with medical services *via* the IMSS-Bienestar subsystem. Before 2003 this population had no access to gratuity for medical services, and the Mexican government created the Social System for Health Protection called “Seguro Popular” through the Fund for Protection against Catastrophic Expenses (FPGC). The FPGC provided monetary resources through a trust to accredited service providers (public and private) at the country level to care for 66 high-cost diseases, including CC, from 2005 to 2018. The study reported the treatment outcome of 38,187 women with CC from 2006 to 2014 covered by FPGC (8). For this analysis, the survival analysis was done in 25,556 women only as 16,619 were excluded (12 with poor-prognosis histology, 8,544 preinvasive diseases, 1,130 recurrent or progression, 1,619 unconfirmed diagnoses, 2,284 and 3,043 had no 5-year follow-up because data on deaths were available until 2019). The results indicate that the FIGO stage distribution was 24.5% for early stages (1A-IB1), 68.5% for locally advanced stages (IB2-IIIB), and 7% for advanced stages (IVA-IVB), with a median age of 51.2 ± 13.8, 49.8 ± 13.6 and 51.6 ± 13.8 respectively. In the multivariate analysis, only the age and clinical stage were significant. For each year of increase in women’s age, the risk of dying increased by 0.3%, while the risk of dying was 2.76 and 5.39 times higher for women with locally advanced and advanced disease, respectively, compared to early stages. Overall survival (OS) at 5 years was 68.5%. The OS analysis by clinical stage was 88% in early stages, 63.9% in locally advanced disease, and 43.6% for advanced disease (8). The results of this study found 5-year survival rates comparable to the reported for other countries, which are 63%, 66%, 67%, and 58.8% for Spain, the USA, Chile, and Colombia, respectively. These outcomes are consistent with others reported by specialized hospitals with oncology departments and cancer centers (5–7, 30). Regarding the factors that affect the survival of patients, a retrospective study that included a cohort of 2,982 women diagnosed with CC, and treated at the INCan, from 2005 to 2015, shows that age at diagnosis is not a prognostic factor for OS or PFS. OS at 5 years in the early stage (FIGO) in women younger than 40 years was 93.4% vs. 92% in women older than 40 years, while in locally advanced disease, it was 62.9% vs. 63.4%, respectively; and advanced disease was 47.5% vs. 46.6%, respectively. The multivariate analysis identified adverse factors contributing to OS and disease-free survival: clinical stage, histological subtype, presence of hydronephrosis, and lymph node involvement (7).

From here, it is clear the importance of providing access to CC treatment in specialized centers to all women, especially those with social disadvantages (8).

## Conclusions

CC is a model of preventable cancer, as demonstrated by the dramatic mortality reduction observed in high-income countries that have successfully implemented screening programs. CC incidence and mortality are closely related to socially disadvantaged women, and such an association remains even in high-income countries. The case of Mexico, an upper-middle-income country, illustrates that CC incidence and mortality are heavily related to the public health system. A universal and efficient health system and a nationwide cancer control program are needed to control CC in Mexico. Despite numerous analyses from epidemiological and medical perspectives, the fact is that this neoplasia still represents a heavy health burden in the world derived from global social and economic inequalities.

## Future perspectives

The eradication of CC remains challenging. From the primary prevention perspective, we must consider statistical models that predict the ability of HPV vaccination to reduce CC mortality. More decades to come are needed to confirm these predictions. Unfortunately, the world population coverage of HPV vaccination is around 15%, which is still far from the threshold of 70% proposed by the WHO. Not all is known regarding HPV vaccination. Some reports have associated vaccination with reductions in the prevalence of HPV infection in unvaccinated women residing at the same geographical location as vaccinated women, presumably by sexual dissemination of these changes. However, vaccine-covered, high-risk HPV types may be replaced by not covered HPV types. In light of these observations, it is not entirely clear what effects vaccine-associated HPV type replacement may be seen in the future (31). Safety issues of HPV vaccination and continued research to ratify the risk-benefit analyzes of these vaccines is desirable (32–34).

Regarding secondary prevention, the Pap test remains widely recommended in most countries though the WHO advocates using HPV-DNA testing primarily or combined with cytology as the primary screening tool for CC, subject to the available resources and infrastructure. The research on alternative simple and effective approaches, such as see-and-treat strategies with visual inspection with acetic acid, must continue, particularly in those countries where HPV testing/Pap smears are unaffordable (35). We must critically analyze the cost-benefit challenge in changing the field of CC screening toward molecular tests (36).

The treatment of CC is perhaps the one facing more problems worldwide. The shortage of trained gynecological surgeons in many countries and regions directly threatens the treatment of patients at early stages (37). For radiotherapy, the situation could be worse. While in high-income countries, one radiotherapy machine is available for every 120,000 people, in middle-income countries, one machine serves over 1 million people and about 5 million people in low-income countries. Cancer patients cannot access radiotherapy in 51 countries, independent territories, and islands (38).

Regarding chemotherapy, low affordability for cancer drugs and medical oncologist specialists seems constant in developing countries (38). Nevertheless, patients from socially disadvantaged conditions attain satisfactory survival rates when they access appropriate cancer care. Therefore, very clever use of resources is required from the view of public health to employ treatments with the highest cost-benefit in settings where resources are insufficient.

## Author contributions

EB: Investigation, Writing-Original Draft. LC-P: Conceptualization, Project administration, Supervision. TC: Investigation. DC-E: Writing-Reviewing and Editing. DR: Supervision. IB: Resources. AD-G: Writing-Original Draft. All authors contributed to the article and approved the submitted version.

## Conflict of interest

The authors declare that the research was conducted in the absence of any commercial or financial relationships that could be construed as a potential conflict of interest.

## Publisher’s note

All claims expressed in this article are solely those of the authors and do not necessarily represent those of their affiliated organizations, or those of the publisher, the editors and the reviewers. Any product that may be evaluated in this article, or claim that may be made by its manufacturer, is not guaranteed or endorsed by the publisher.
